# Enhancement of autonomic and psychomotor arousal by exposures to blue wavelength light: importance of both absolute and relative contents of melanopic component

**DOI:** 10.1186/s40101-017-0126-x

**Published:** 2017-01-31

**Authors:** Emi Yuda, Hiroki Ogasawara, Yutaka Yoshida, Junichiro Hayano

**Affiliations:** Department of Medical Education, Nagoya City University Graduate School of Medical Sciences, Nagoya, 467-8602 Japan

**Keywords:** Arousal, Blue light, Heart rate variability, Intrinsically photosensitive retinal ganglion cell, Melanopic component, Melanopsin, Non-image forming vision, Organic light-emitting diode, Psychomotor vigilance

## Abstract

**Background:**

Blue light containing rich melanopsin-stimulating (melanopic) component has been reported to enhance arousal level, but it is unclear whether the determinant of the effects is the absolute or relative content of melanopic component. We compared the autonomic and psychomotor arousal effects of melanopic-enriched blue light of organic light-emitting diode (OLED) with those of OLED lights with lesser absolute amount of melanopic component (green light) and with greater absolute but lesser relative content (white light).

**Methods:**

Using a ceiling light consisting of 120 panels (55 × 55 mm square) of OLED modules with adjustable color and brightness, we examined the effects of blue, green, and white lights (melanopic photon flux densities, 0.23, 0.14, and 0.38 μmol/m^2^/s and its relative content ratios, 72, 17, and 14%, respectively) on heart rate variability (HRV) during exposures and on the performance of psychomotor vigilance test (PVT) after exposures in ten healthy subjects with normal color vision. For each of the three colors, five consecutive 10-min sessions of light exposures were performed in the supine position, interleaved by four 10-min intervals during which 5-min PVT was performed under usual fluorescent light in sitting position. Low-frequency (LF, 0.04–0.15 Hz) and high-frequency (HF, 0.15–0.40 Hz) power and LF-to-HF ratio (LF/HF) of HRV during light exposures and reaction time (RT) and minor lapse (RT >500 ms) of PVT were analyzed.

**Results:**

Heart rate was higher and the HF power reflecting autonomic resting was lower during exposures to the blue light than the green and white lights, while LF/HF did not differ significantly. Also, the number of minor lapse and the variation of reaction time reflecting decreased vigilance were lower after exposures to the blue light than the green light.

**Conclusions:**

The effects of blue OLED light for maintaining autonomic and psychomotor arousal levels depend on both absolute and relative contents of melanopic component in the light.

## Introduction

In humans, light is a crucial source of stimuli that enhance the arousal systems and entrain their rhythms to those of the external environments. These actions of light can be elicited independently of the functions of image-forming vision and have greater sensitivity to blue wavelength light [1–4]. This is presumably resulted from the involvement of intrinsically photosensitive retinal ganglion cells (ipRGCs) expressing the photopigment melanopsin, and which are sensitive to blue wavelength around 480 nm [5–9]. These facts suggest the possibility to develop new lighting sources for manipulating arousal levels by adjusting the content of melanopsin-stimulating (melanopic) component in accordance with desired activities such as work, study, rest, and sleep [1, 3]. Earlier studies on the physiological effects of blue lights, however, have used monochromatic lights with sharp-single-peak spectra or polychromatic fluorescent lights with complicate multiple-peak spectra [10, 11]. Consequently, it is still unclear whether the determinant of arousal effects of blue light is the absolute amount of melanopic component or its relative content ratio, which seems important to ensure both safety and efficacy of new lighting sources.

In this study, we developed an interior ceiling light system using organic light-emitting diode (OLED), which provides non-glaring surface illumination with rich color rendering properties. Compared with conventional light-emitting diode and fluorescent lamps, OLED can generate lights with broader spectra including those close to natural lights. Particularly, blue OLED light contains rich melanopic component whose spectral distribution is close to that reported for the melanopic efficiency function [12, 13]. Using this lighting system, we examined the effects of OLED blue light on autonomic and psychomotor arousal levels and compared them with those of other OLED lights with a lesser absolute amount of melanopic component (green light) and with a greater absolute amount but a lesser relative content ratio of melanopic component (white light). We assessed the autonomic and psychomotor arousal levels by the analysis of heart rate variability (HRV) and the performance of psychomotor vigilance test (PVT), respectively. Additionally, we performed a supplementary study to examine pupillary light reflex to these three lights.

## Methods

### Subjects

The subjects of the main study were recruited with the following inclusion criteria: healthy men or women who (1) were between 20 and 40 years old, (2) had normal color vision, (3) were not taking any medications for >2 weeks, and (4) displayed a normal sinus rhythm on electrocardiogram (ECG) at rest. There were ten applicants (mean age ± SD, 26 ± 5 year, four females) who met the inclusion criteria and all participated in the main study.

Additionally, seven subjects (41 ± 9 year, three females) were recruited for the supplementary study of pupillary light reflex with the same inclusion criteria except for the age range, which was between 20 and 55 years old. All of these subjects gave their written informed consent to participate in this study.

### OLED lighting device

**Fig. 1:**
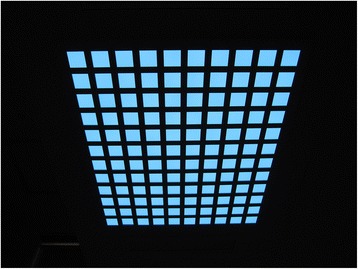
Ceiling light device with organic light-emitting diode (OLED). The device consists of 120 OLED panels (VELVE OLED lighting module with adjustable red-green-blue color and brightness, 55 × 55 mm square, Mitsubishi Chemical Pioneer OLED Lighting Corporation, Tokyo, Japan) that are aligned in a 10 × 12 reticular pattern

**Fig. 2:**
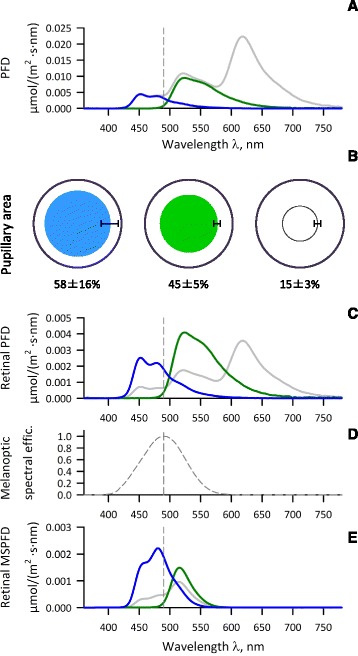
Photon flux density (PFD) spectra of colored OLED lights and relative papillary area measured during exposure to these lights in the supplementary study. **a** PFD spectra of blue, green, and white lights measured at the place of the eyes of subjects lying on a bed right under the ceiling light. **b** Average relative pupillary area measured during exposure to the lights in seven subjects in the supplementary study. *Error bars* indicate the range of ± SD of relative diameters. **c** Estimated PFD spectra of lights reaching the retina calculated from the relative pupillary areas during exposure to the lights. **d** Melanopic spectral efficiency curve adjusted for human pre-receptoral filtering generated from the data in reference [13]. *Vertical dashed lines* in both panels indicate the position of *λ*_max_ of the adjusted melanoptic spectral efficiency (490 nm). **e** Estimated MSPFD spectra of lights reaching the retina calculated from the relative pupillary areas during exposure to the lights

We have developed a ceiling light system for the present study (Fig. 1). The device consisted of 120 OLED panels (VELVE OLED Lighting Module with adjustable red-green-blue color and brightness, 55 × 55 mm square, Mitsubishi Chemical Pioneer OLED Lighting Corporation, Tokyo, Japan) that were aligned in a 10 × 12 reticular pattern. The device was placed right above the eyes of subjects lying on a bed in the supine position. We adjusted the lighting device so that they emitted three kinds of colored lights (blue, green, and white); Fig. 2a shows the spectrum of photon flux density (PFD) of the three colored lights, and Table 1 shows their optical characteristics. The melanopsin-stimulating photon flux densities (MSPFDs) calculated from the melanoptic spectral efficiency curve adjusted for the effect of human pre-receptoral filtering [8, 12, 13] were 0.23, 0.14, and 0.38 μmol/m^2^/s and the relative content ratios were 72, 17, and 14% for these blue, green, and white lights, respectively.

**Table 1:** Characteristics of organic light-emitting diode (OLED) lights used in this study

	Blue	Green	White
Illuminance, lx	13	91	158
Irradiance, W/m^2^	0.08	0.18	0.54
Chromaticity (x, y)	0.14, 0.16	0.33, 0.62	0.44, 0.41
Weighted mean wavelength, nm	483	555	594
PFD, μmol/m^2^/s	0.32	0.83	2.62
MSPFD, μmol/m^2^/s^a^	0.23	0.14	0.38
Relative MSPFD, %	72	17	14

^a^Calculated from melanoptic spectral efficiency adjusted for human pre-receptoral filtering [12, 13]

*MSPFD* melanopsin-stimulating photon flux density, *PFD* photon flux density

### Measurements

In the main study, lead II ECG were recorded continuously with an eight-channel bioelectric amplifier (Biotop mini, East Medic Corporation, Kanazawa, Japan), digitized at 500 Hz with an analog-to-digital converter (AIO-163202FX-USB, CONTEC Corporation, Osaka, Japan), and stored in a hard disk of a personal computer.

For the PVT, we used a validated software called PC-PVT [14], whose free software was downloaded from http://bhsai.org/downloads/pc-pvt/ and installed in a notebook personal computer (Let’s note CF-S10, Panasonic Co., Osaka, Japan). The anticipation was set at 100 ms, deadline at 65,000 ms, and minor lapse at 500 ms. PVT measures sustained or vigilant attention by recording reaction time (RT) to visual stimuli occurring at random inter-stimulus intervals. The PVT software presented abruptly a time counter on computer display that incremented the number at every 1 ms. In this study, the total trial time was set at 300 s, during which 46 to 51 stimuli were presented. According to the earlier studies [15], we measured the number of minor lapse frequency (RT ≥500 ms) with transformation $\left(\sqrt{x}+\sqrt{x+1}\right)$, fastest and slowest tenth percentile RT, and difference between fastest and slowest RT.

In the supplementary study, pupillary diameters in darkness as well as during exposure to blue, green, and white lights were measured with an open-type electric pupillometer (View Shot, FP-10000II, T.M.I. Co., Niiza City, Saitama, Japan) and stored in a hard disk of a personal computer.

### Study protocols

Subjects were instructed not to consume food or beverages containing caffeine or alcohol after 21:00 the previous night and to take >7 h of sleep. All studies were performed between 09:30 and 14:00 in a calm, light-shielded, and air-conditioned (24 ± 2 °C) laboratory more than 2 h after a light meal.

**Fig. 3:**
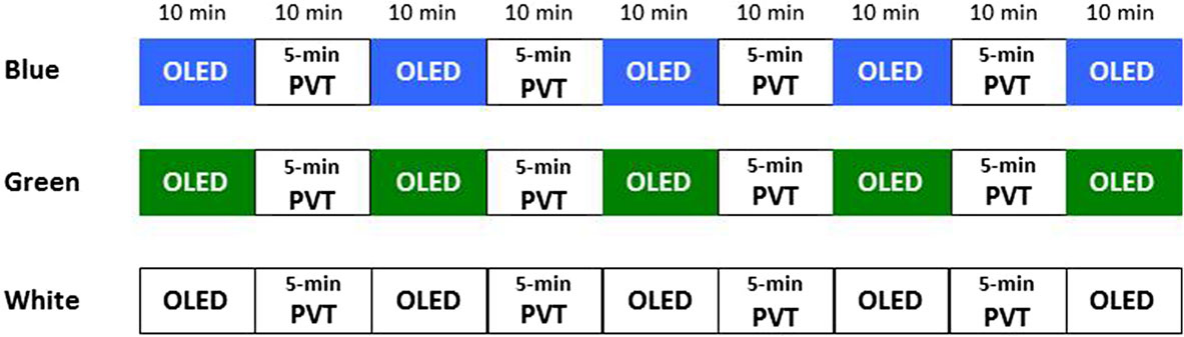
Experimental protocol of the main study. The series of OLED light exposure sessions with blue, green, and white lights were performed in all subjects with orders counterbalanced across subjects

Figure 3 shows the protocol of the main study. All of the ten subjects performed three series of sessions with different color lights (blue, green, and white) on different days with ≥1 week of washout period. Within each subject, the experiments with different color lights were performed at exactly the same clock time to prevent the circadian rhythm of autonomic functions and alertness from affecting the results. The orders were counterbalanced across subjects. On each day, subjects lied on the bed in the supine position, so that their eyes were right below the OLED device. During each OLED lighting session, the subjects were instructed to keep their eyes open and to gaze at the OLED panels for 10 min. We performed the measurements in the supine position in order to prevent the slight difference in body posture (such as the angle of upper body at sitting posture) from affecting the cardiac autonomic function [16].

In each PVT session, subjects sat to a table for 10 min, during which 5-min PVT was performed under usual white fluorescent light (desktop illuminance, 300 lx) in order to prevent the colored lightings from affecting the visibility of the PVT screen. The session was repeated four times following each OLED light exposure session.

The supplementary study was performed for examining pupillary light reflex to blue, green, and white lights on a day separated from the main study. The subjects lied on the bed in the supine position without glasses if they were using so that their eyes were right below the OLED device. The electronic pupillometer was secured vertically in front of subjects’ right eye so that they were able to gaze at OLED panels through the pupillometer. During measurement, the subjects were instructed to keep their eyes open and to gaze at the OLED panels with their both eyes. The pupillary diameter was measured continuously in >3-min darkness for a dark adapted baseline followed by light reflex after lighting until the diameter leaches to a steady state photoequilibrium after an initial transient response [17]. The pupillary reflexes to all three colors were examined in all subjects in the orders counterbalanced across subjects.

### Data analyses

Digitized ECG data were analyzed off-line on a personal computer. The temporal positions of all QRS waves were detected with a fast-peak detection algorithm. After all errors in the detection of QRS waves were edited, time series of the R-R interval were obtained. The R-R interval time series thus obtained were analyzed separately for five sessions with three different colors of light. For each data segment, frequency domain analyses of the HRV were performed with fast Fourier transformation (FFT) with the original software in our laboratory [18]. Briefly, R-R interval time series were interpolated with a step function only using interval data consisting of consecutive QRS waves in sinus rhythm, resampled at 1024 equidistant time points for 10-min data segments, filtered with a Hanning window, and converted into frequency domain by FFT. After correcting for the losses of variance resulting from the sampling and filtering processes, the power of the low-frequency (LF, 0.04–0.15 Hz) and the high-frequency (HF, 0.15–0.40 Hz) components were computed. The power of these components was transformed into the natural logarithmic value.

For the PVT data, RT was averaged and minor lapse frequency was calculated as the percentage of the number of minor lapses in the total number of stimuli over each PVT session.

For the pupillometric data, relative pupillary area for each color was calculated with dividing the pupillary area at steady state photoequilibrium by the area of dark adapted baseline in individual subjects.

### Statistical analysis

Statistical analyses system version 9.4 (SAS institute Inc., Cary, NC, USA) was used for the statistical analysis. Our interests were to clarify whether the autonomic neural activities during light exposure and the performance of PVT after the light exposure differ among blue, green, and white OLED lights. For these purposes, we used the mixed-model analyses of variance (ANOVA) for repeated measures with color of light (blue, green, and white), session, and interaction between color and session, and sex as the fixed effects and subject as random effect. *P* < 0.05 was considered to be statistically significant, and Bonferroni adjustment was used to keep type 1 error level in multiple comparisons.

## Results

**Fig. 4:**
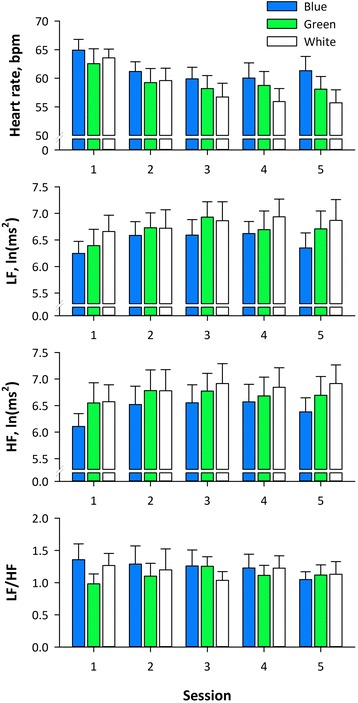
Mean heart rate and heart rate variability indices during five sessions of blue, green, and white light exposures in ten subjects in the main study. *Error bars* indicate standard error of the means. *HF* high-frequency component, *LF* low-frequency component, *LF/HF* LF-to-HF ratio in power

Figure 4 shows the heart rate and HRV indices during colored OLED lighting sessions. Repeated measures ANOVA showed significant effects of light color and session on heart rate, HF power, and LF power, while there was no significant interaction between light color and session and no significant effect of sex on any of these measures (Table 2). Multiple comparisons revealed that heart rate was higher and HF power was lower during exposure to blue light than green and white lights, while there was no difference between green and white lights.

**Table 2:** Repeated measures ANOVA of the effects of light color, session, and sex on heart rate and HRV indices

	Fixed effects								Post hoc multiple comparisons^a^					
	Color		Session		Color × session		Sex		Blue vs green		Blue vs white		Green vs white	
	*F*_2,126_	*P*	*F*_4,126_	*P*	*F*_8,126_	*P*	*F*_1,126_	*P*	*t*_126_	*P*	*t*_126_	*P*	*t*_126_	*P*
Heart rate	8.56	0.0003	10.83	<0.0001	0.72	0.6	0.71	0.4	2.71	0.02	4.06	0.0003	1.36	0.5
LF power	6.64	0.001	2.82	0.02	0.47	0.8	0.28	0.5	−2.31	0.06	−3.59	0.004	−1.28	0.6
HF power	13.16	<0.0001	3.68	0.007	0.45	0.8	0.03	0.8	−3.56	0.001	−4.98	<0.0001	−1.42	0.4
LF/HF	0.64	0.5	0.16	0.8	0.44	0.8	0.29	0.5	1.13	0.7	0.60	1.0	−0.54	1.0

^a^Bonferroni adjustment

*HF* high-frequency component, *LF* low-frequency component, *LF/HF* LF-to-HF ratio in power

**Fig. 5:**
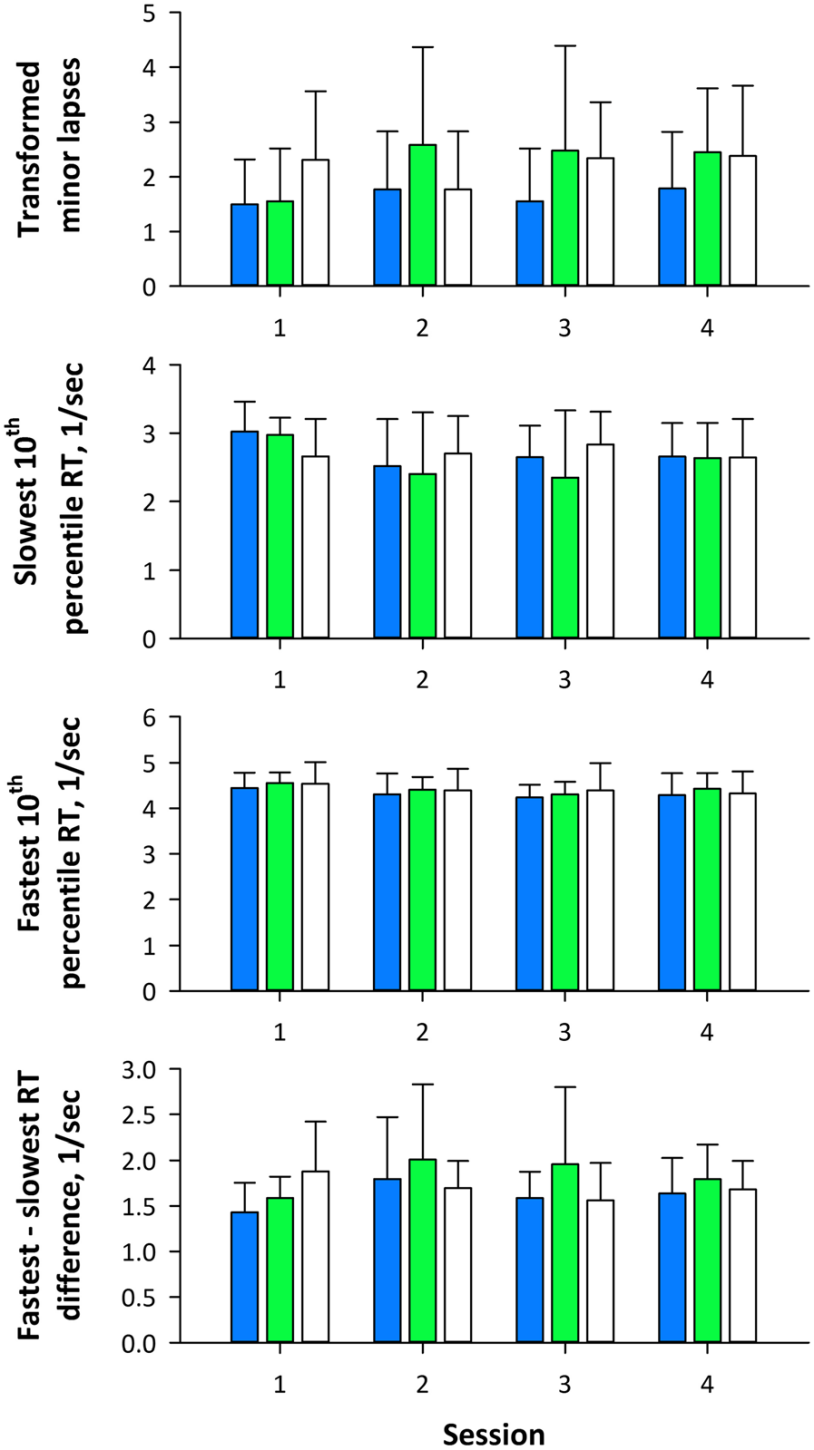
Transformed minor lapses, slowest and fastest tenth percentile reaction times (RT), and fastest-slowest RT difference during four sessions of psychomotor vigilance test (PVT) after blue, green, and white light exposures in ten subjects in the main study. *Error bars* indicate standard error of the means

Figure 5 shows the minor lapse and RTs during PVT session. Repeated measures ANOVA showed significant effect of light color on the transformed number of minor lapse and the difference between fastest and slowest RTs (Table 3). Multiple comparisons revealed that the number of minor lapse and the fastest-slowest RT difference was lower after exposure to blue light than green lights, while there was no difference between blue and white or between green and white colors.

**Table 3:** Repeated measures ANOVA of the effects of light color, session, and sex on psychomotor vigilance test (PVT)

	Fixed effects								Post hoc multiple comparisons^a^					
	Color		Session		Color × session		Sex		Blue vs green		Blue vs white		Green vs white	
	*F*_2,99_	*P*	*F*_3,99_	*P*	*F*_6,99_	*P*	*F*_1,99_	*P*	*t*_99_	*P*	*t*_99_	*P*	*t*_99_	*P*
Transformed minor lapse	3.72	0.02	0.81	0.4	0.96	0.4	1.69	0.1	−2.49	0.04	−2.22	0.08	0.27	1.0
Slowest 10% RT	0.89	0.4	3.15	0.02	1.59	0.1	0.79	0.3	1.16	0.7	0.01	1.0	−1.15	0.7
Fastest 10% RT	1.86	0.1	3.33	0.02	0.22	0.9	0.33	0.5	−1.78	0.2	−1.53	0.3	0.26	1.0
Fastest-slowest RT difference	3.29	0.04	1.37	0.2	1.78	0.1	0.27	0.6	−2.55	0.03	−1.03	0.9	1.52	0.3

^a^Bonferroni adjustment

*RT* reaction time

In the supplementary study, the pupils were contracted with lights as an initial transient response followed by gradual recovery and leached to a steady state photoequilibrium within 2 min in all subjects. The averages ± SD of relative pupillary area at the photoequilibrium to the dark adapted baseline was 58 ± 16%, 45 ± 5%, and 15 ± 3% for blue, green, and white lights, respectively. When these ratios were applied to MSPFD of each color of light shown in Table 1, the amounts (MSPFD) of melanoptic component estimated to reach the retina were 0.13, 0.062, and 0.056 μmol/m^2^/s for blue, green, and white lights, respectively.

## Discussion

To clarify whether the arousal effects of blue-enriched light depends on the absolute amount of melanopic component or its relative content ratio in the light, we compared the autonomic and psychomotor arousal effects among melanopic-enriched OLED blue light, OLED green lights containing lesser absolute amount of melanopic component, and OLED white light containing greater absolute but lesser relative content. We observed that heart rate was higher and the HF power was lower during exposure to the blue light than exposures to green and white lights. Also, we observed fewer minor lapse and lower fastest-lowest RT difference after exposure to blue light than exposure to green light. Because the HF component of HRV reflects the level of cardiorespiratory resting function [19, 20], the lower HF power observed during exposures to blue light is indicative of sustained higher arousal. Also, the fewer minor lapse and the smaller variance in RT of PVT indicate sustained higher alertness. The findings of this study therefore suggest the importance of relative content ratio as well as absolute amount of melanopic component to elicit sustained arousal and alertness during and after exposures to the light.

Our observations of the sustained arousal and alertness effects of blue-enriched light are in agreement with several earlier reports. In a study of healthy male subjects, Cajochen et al. [1] have compared the two monochromatic lights at 460 and 550 nm. They reported occurrence of greater melatonin suppression, greater alerting response, and higher core body temperature with a 2-h exposure to light at 460 nm than at 550 nm. In a previous study in health young subjects, we have compared the acute effects on HRV of 6-min exposures to blue, green, and red OLED lights with a weighted mean wavelength of 482, 553, and 640 nm, respectively [21]. We observed a greater suppression of HF power with the blue OLED light than the green and red OLED lights. Chellappa et al. [3] have compared the effects of three polychromatic lights with correlated color temperatures at 6500, 2500, and 3000 K and with the same illuminance of 40 lx in healthy male subjects. They observed that light at 6500 K that produced higher spectral power between 420 and 520 nm induced greater melatonin suppression, higher subjective alertness, and faster RT in PVT. From these earlier findings, however, it is unclear whether the determinant of the effects of blue light is the absolute amount of melanopic component or its relative content ratio.

The differences in the effects between blue and green OLED lights are explained simply by the difference in absolute amount of melanopic component. This is also supported by the findings of our earlier study [21], in which we used the same OLED modules as in the present study and observed a significant suppression of HF power only during exposure to a 10-lx blue OLED light but not during exposure to 5- or 2-lx blue OLED lights. In contrast, the mechanisms for the differences in the effects between blue and white OLED lights may be equivocal, although the results suggest the importance of relative content ratio of melanopic component. To investigate the possible roles of pupillary light reflex for these findings, we performed the supplementary study. The average relative pupillary areas at photoequilibrium to dark adaptive baseline in health subjects were 58, 45, and 15% for the blue, green, and white OLED lights, respectively (Fig. 2). If these ratios were applicable to MSPFD of these lights, the estimated amount of melanoptic component reaching the retina would be 0.13, 0.062, and 0.056 μmol/m^2^/s, which seems consistent with the effects of autonomic and psychomotor arousal of the three OLED lights. This discussion is only speculative, however, because the main and supplementary studies were performed separately with different subjects’ groups.

To our knowledge, this is the first study to report the effects of ambient OLED colored lights on the arousal levels. OLED has rich color rendering properties and provides non-glaring lights with a smooth broader spectrum. Particularly, the photon flux spectral density of blue OLED light is close to that reported for the melanopic efficiency function [12, 13]. These features of OLED allowed us to generate three types of light with desired absolute and relative components of melanopic component. OLED is expected to be used as a new lighting source at home, workplace, and healthcare environments. Our findings of the arousal and alertness effects and their determinants seem important to ensure both safety and efficacy of OLED devices when they are used as new lighting sources.

### Limitations

This study has several limitations. First, because we used only OLED for lighting source in this study, our findings may not be extended to other lighting sources. Particularly, because we compared monochromatic (blue and green) and polychromatic (white) lights, it is unclear whether the different autonomic effects were caused by the differences in physical property of light or by those in psychological effects of colors. Also, the light intensity of blue light used in this study was very low (13 lx illuminance and 0.18 W/m^2^ irradiance). Because the light intensity is known to have a strong effect on melatonin secretion [10, 11], further studies with differing light intensity are required. Second, because we did not collect baseline control data during the dark, we cannot exclude the influence of possible differences in baseline autonomic functions and psychomotor performance among the three lights. Also, because the subjects included four females, their autonomic functions may have had been influenced by menstrual cycle of experiment days [22]. The results of statistical analyses, however, revealed no significant effect of sex on any of the measures of this study. Also, these effects, if any, would have been reduced statistically, because the order of the three lights was counterbalanced across subjects. Third, because we did not measure melatonin secretion, we were unable to determine whether the OLED lights affect the entrainment to environmental light-dark cycles or not. Also, we cannot ensure that the effects of OLED lights on arousal levels were mediated by melanopsin-dependent non-image forming responses. Fourth, although this study aimed at examining acute arousal effects of OLED lights, the analyses of long-term benefits are also needed in the future studies. Given the application to real workplace settings, it is important to examine the after-effects on autonomic and psychomotor functions. Also, for practical applications, further investigations with other lighting devices, including portable device and light bulbs that can be used with existing lighting equipment, are also necessary. Finally, this is a preliminary study with a small sample size. To determine the effects of gender and aging on the results, future studies are necessary.

## Conclusions

To clarify whether the arousal effects of blue-enriched light depends on the absolute amount of melanopic component or its relative content ratio in the light, we compared the autonomic and psychomotor arousal effects of melanopic-enriched blue light with OLED lights with different absolute amount and relative content of melanopic component. Our findings suggest the importance of relative content ratio as well as absolute amount of melanopic component to elicit sustained arousal and alertness during and after exposures to the light.

## Abbreviations

ECG: Electrocardiogram
FFT: Fast Fourier transformation
HF: High frequency
HRV: Heart rate variability
ipRGC: Intrinsically photosensitive retinal ganglion cell
LF: Low frequency
LF/HF: LF-to-HF ratio
MSPFD: Melanopsin-stimulating photon flux density
OLED: Organic light-emitting diode
PFD: Photon flux density
PVT: Psychomotor vigilance test
RT: Reaction time
